# Optical-Based Foot Plantar Pressure Measurement System for Potential Application in Human Postural Control Measurement and Person Identification †

**DOI:** 10.3390/s21134437

**Published:** 2021-06-28

**Authors:** Tanapon Keatsamarn, Sarinporn Visitsattapongse, Hisayuki Aoyama, Chuchart Pintavirooj

**Affiliations:** 1 King Mongkut’s Institute of Technology Ladkrabang, School of Engineering, Bangkok 10520, Thailand; 60601222@kmitl.ac.th (T.K.); sarinporn.vi@kmitl.ac.th (S.V.); 2 Faculty of Engineering, The University of Electro-Communications, Tokyo 182-8585, Japan; aoyamaxer@gmail.com

**Keywords:** human postural balance, gait analysis, biometrics, foot pressure

## Abstract

Plantar pressure, the pressure exerted between the sole and the supporting surface, has great potentialities in various research fields, including footwear design, biometrics, gait analysis and the assessment of patients with diabetes. This research designs an optical-based foot plantar pressure measurement system aimed for human postural control and person identification. The proposed system consists of digital cameras installed underneath an acrylic plate covered by glossy white paper and mounted with LED strips along the side of the plate. When the light is emitted from the LED stripes, it deflects the digital cameras due to the pressure exerted between the glossy white paper and the acrylic plate. In this way, the cameras generate color-coded plantar pressure images of the subject standing on the acrylic-top platform. Our proposed system performs personal identification and postural control by extracting static and dynamic features from the generated plantar pressure images. Plantar pressure images were collected from 90 individuals (40 males, 50 females) to develop and evaluate the proposed system. In posture balance evaluation, we propose the use of a posture balance index that contains both magnitude and directional information about human posture balance control. For person identification, the experimental results show that our proposed system can achieve promising results, showing an area under the receiver operating characteristic (ROC) curve of 0.98515 (98.515%), an equal error rate (EER) of 5.8687%, and efficiency of 98.515%.

## 1. Introduction

Plantar pressure measurement provides crucial information about the human health condition, the dynamic and static structure of the human body, and foot functionality [1]. Significant advancements have been made in developing up-to-date measurement technologies for various applications of plantar pressure measurement to help understand general behavior and/or pathologies related to human motion [2,3].

Human balance analysis, which is an application of plantar pressure, has been an active research field for a decade. In some diseases, such as Parkinson’s disease, a deficiency in specific neurotransmitters impairs motor control in the brain. As a result, patients lose control of their motion. In this situation, plantar pressure-based balance analysis can be used to estimate the degree of motor control degeneration. Hernandez et al. [4] analyzed human postural control using a force plate to measure the center of pressure (COP) and velocity autocorrelation function (COP-VAF). They performed their experiments in eleven healthy young participants under quiet unipedal or bipedal standing conditions with eyes either opened or closed. Hernandez et al. used a commercial ground-level six-channel force plate (AccuGait, AMTI, Watertown, MA, USA), which is quite expensive. Ma and Hu [5] proposed the use of a thermal imaging system and insole pressure sensors. They developed novel algorithms to extract the leg skeletons from the thermal images and implement motion signals for human postural control analysis. The limitation of the technique proposed by Ma and Hu is the acquisition system, which uses a thermal camera that requires a high sampling rate and hence is costly. Kharboutly et al. [6] presented the design of a multiple-axis robotic platform for postural stability analysis. Standing on the platform, the subject is evaluated for various dynamic posture stability control features, including (i) sagittal tilt angle, (ii) frontal tilt angle, (iii) vertical rotation angle, and (iv) translation. The designed robotic platform of Kharboutly et al. is a very complicated structure and hence its construction is expensive. Ma et al. [7] designed an omni-directional platform for balance analysis. Measuring the COP, the system is equipped with force and inertial sensors intended for the evaluation of human posture for static and dynamic equilibrium analysis of a human subject standing on it. The system is also capable of applying balance disturbance to measure the subject’s balance efficiency. The advantage of Ma et al.’s platform is its compactness and structure, which offer a better sense of comfort and safety when used in small spaces such as therapists’ offices or hospitals. Gopalai and Senanayake [8] used four force-sensing resistors (FCR) attached to a platform to assess balance stability in real time. Stability was measured in eyes open (EO) and eyes closed (EC) states for flat ground (static) and unstable (dynamic) conditions. A drawback of Senanayake’s design is the low resolution due to the use of only four force-sensing sensors. Other related research works for human postural balance analysis are referred to [9,10].

Another application of plantar pressure, gait analysis, has also captured researchers’ attention during the last decade [11,12,13,14]. Abnormal or asymmetrical walking patterns lasting for a long period of time can lead to health problems including musculoskeletal disorders, cardiovascular diseases, and depression. Gait analysis can then be used to determine the level of gait abnormality, evaluate potential treatments, and treat the abnormality. The modern gait analysis technology uses several infrared markers attached to the patient’s joints and captures images with several cameras located around a walkway or a treadmill. The trajectory of each marker can be calculated and displayed in three dimensions on the monitor [11]. Gait analysis using a 3D acquisition system is quite expensive. Many researchers have used alternative methods for gait analysis. In-shoe plantar measurement systems are by far the most used alternative tool for gait analysis measurement. The plantar pressure sensor includes a capacitive sensor, a resistive sensor, a piezoelectric sensor, a textile sensor [12], an air-filled pad with an elastomeric pillar array [13], and an optical fiber sensor [15]. Ramirez-Bautista and Huerta-Ruelas [12] provided a good review of shoe plantar measurement systems. Ferreira et al. [16] attached white light-emitting diodes (LEDs) on several points of an individual’s body while capturing images during his locomotion in front of a webcam. The gait characteristics were extracted using a priori anatomical images. The attachment of only four white light-emitting diodes (LEDs) per one in the technique proposed by Ferreira et al. limits the accuracy of the gait analysis. Park et al. [17] designed a soft robotic ankle foot orthosis for post-stroke patients that is capable of real-time gait analysis for rehabilitation. The device also contains a wearable gait-sensing module for measuring the leg trajectory and the foot pressures in real time for feedback control. One of the drawbacks of the soft wearable gait-sensing module of Park et al. is that the wearing of the module could affect the natural movement the body. Refai et al. [18] developed a remote real-time monitoring system for dynamic gait and balance performance using force and moment sensors and inertial measurement units (IMU) installed in the shoe. Three-dimensional force and moment are estimated using a linear regression model. Refai et al.’s research work is customized for specific subjects and hence requires a calibration phase that involves observing the subject walking for a brief period. A generic model that can be applied to any subject is envisaged for future work. A Kinect sensor, which includes a camera and a depth sensor, has been used recently for gait analysis [19,20,21] due to its capability of providing a 3D virtual skeleton of the body. Stone and Skubic [19,20] were the pioneers who used Kinect for clinical gait analysis. They used a depth image to locate objects at the height of 50 cm or less in their work. They applied a volume of these objects to infer whether the left or the right foot was touching the ground. The method proposed by Stone and Skubic requires no markers or battery-powered sensors, and instead relies on a single, inexpensive, and widely available 3D sensor. Gabel et al. [21] used information from the entire body captured from Kinect to measure stride intervals more accurately. Although Kinect-based gait analysis is a promising technique, it is sensitive to occlusion, when capturing the subject from the side view, which still limits the adoption of the device.

The gait pattern of an individual is quite unique and can be used for biometrics. Gait classification and recognition are active research fields that have recently captured researchers’ attention [22,23,24,25,26,27]. Various techniques have been used for gait classification and recognition that can be roughly classified into two categories: (i) video-based and (ii) pressure sensor-based methods. In video-based techniques, a camera (2D or 3D) is used to capture human gait. The captured image frames are then processed by computer vision software to recognize or to identify persons. Sinha et al. [22] used Kinect to extract area and distance features for real-time person recognition. The area feature is defined as the area spanning the lower and upper parts of the body, where the distance features are the relative distances between body and centroid and the centroids formed by the joints of upper and lower limbs. The proposed technique provides accuracy of only 90%. The use of multiple Kinect cameras has been suggested to improve the performance. Yamada et al. [23] proposed the use of a real-time multi-line light detection and ranging (LiDAR) camera, which can obtain three-dimensional range information about the target, for person identification. The collected pedestrian data of the subject are trained and classified by a convolutional neural network (CNN). Evaluated on 30 subjects, the technique can achieve a classification performance of only 60%. Researchers have also considered pressure-based gait classification and recognition. Nguyen et al. [24] used smart shoes that contain eight plantar pressure sensors each, to classify five ambulatory activities in real time, i.e., level ground, incline descent, incline ascent, stair descent, and stair ascent walking. The drawback of the method proposed by Nguyen et al. is the low resolution due to the limited number of installed sensors. A low-cost intelligent carpet system was proposed by Agrawal et al. [25] to recognize the normal walking signature of an individual. The carpet consists of activated, nonwoven, carbon material sandwiched between a pair of cross-aligned conductive wires to form in situ piezoresistive sensors. Takeda et al. [26] developed a biometric personal authentication system by detecting one step foot pressure distribution change using a load distribution sensor. A 6.1% EER (equal error rate) was reported. Connor [27] used an underfoot pressure sensor for a fully fledged gait biometric modality. The study was tested in different case scenarios, including barefoot gait recognition and shoe foot recognition when the shoe was used in training. The overall gait recognition results were encouraging and, in the barefoot case, approached the level of high-performance fingerprint and iris biometrics, with an EER of 2.13%.

Optical-based plantar pressure measurement is another promising method that can be used to estimate the pressure distribution of the sole [28]. The system consists of a camera installed underneath the translucent platform. To measure plantar pressure, the subject stands on the translucent platform while the camera captures the plantar image. Due to the use of a camera, the optical-based system has one drawback, i.e., the thickness of the system, which depends upon the focal length of the camera. To decrease the thickness, some systems use a reflective mirror placed at a 45-degree orientation below the platform or use a camera with a small focal length. In this research [29], we propose a design for a low-cost, easy-to-implement, multi-camera, optical-based plantar pressure measurement system. The system consists of a series of digital cameras installed underneath an acrylic-top platform. Light from an LED strip mounted along the side of the acrylic plate is deflected by the pressure exerted between the glossy white paper installed on top of the acrylic, and the digital cameras provide a color-coded plantar pressure image of the subject standing on the platform. We demonstrate the application of our optical-based plantar pressure measurement for human posture balance control evaluation and person identification analysis. In human posture balance control evaluation, we define the new evaluation parameters based on the ellipsoidal fit to the trajectory of the center of pressure, which contains both the magnitude and direction of the human posture balance control. For person identification applications, we propose the use of a hybrid identification/recognition feature, which combines a static plantar pressure feature and gaiting plantar pressure feature.

The paper is organized as follows. Section 2 explains the design of the optical-based plantar pressure measurement system. Section 3 is devoted to the human posture balance measurement. Section 4 explains personal identification and recognition. The discussion and conclusions are presented in Section 5 and Section 6, respectively.

## 2. Optical-Based Plantar Pressure Measurement System

**Figure 1:**
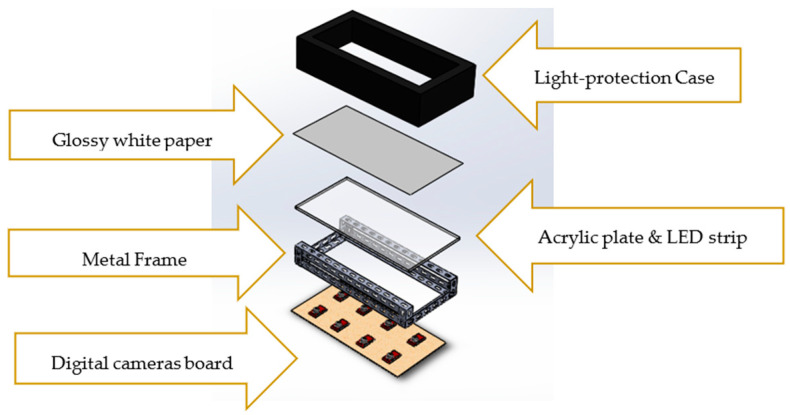
The designed optical-based plantar pressure measurement platform.

Our designed optical-based plantar pressure measurement platform is shown in Figure 1, and it consists of 5 parts: (i) light protection case, (ii) glossy white paper, (iii) acrylic plate with LED strips, (iv) metal frame, and (v) digital camera board. The subject will step on the top of the platform covered with the acrylic plate and glossy white paper to measure their foot plantar pressure. Light from the LED strip installed around the acrylic plate will be deflected towards the series of cameras installed underneath due to the pressure exerted between the paper and acrylic surface. The captured images will then be mosaicked to obtain the color-coded plantar pressure image. In this study, we use a series of USB cameras for two reasons. First, we wish to increase the captured area of the plantar pressure image, especially for dynamic plantar pressure acquisition or for capturing the subject walking. Second, we can reduce the height of the platform and hence reduce any possibilities of bodily injury. All the components are covered by a light protection case to prevent ambient light interference during the acquisition process. Our optical-based plantar pressure measurement system can also be cascaded to increase the walking path for better gaiting plantar pressure.

### 2.1. Foot Plantar Pressure Acquisition Process

The acquisition process for the foot plantar pressure image is divided into four steps, including (i) distortion correction, (ii) intensity offset and contrast offset adjustment, (iii) mosaicking process, and (iv) smoothing of the transition region. Detailed descriptions of each step are included in this section.

#### 2.1.1. Distortion Correction

Prior to the mosaicking process of the plantar pressure images, it is crucial to correct the image distortion due to the digital camera’s intrinsic properties. There are two types of non-linear camera distortion, which are barrel and pin-cushion distortion. To estimate the non-linear correction parameters, the standard camera calibration process is applied [30] by taking a series of images of a chessboard. The acquired parameters are then used for correcting image distortion with OpenCV command [31].

#### 2.1.2. Intensity Offset and Contrast Offset Adjustment

The acquisition process uses multiple digital cameras. These cameras must have the following properties: (i) identical intrinsic properties, (ii) same extrinsic properties except translation properties, and (iii) the same intensity and contrast properties. The identical intrinsic properties can be ensured by using the same USB camera model. The same extrinsic properties can be ensured by carefully aligning the camera. The offset of intensity and contrast properties can be solved using the following process.

- Intensity Offset Adjustment
  To ensure that the camera has the same intensity or brightness level, we compute the mean intensity in each camera and then find the difference in the average intensity. Then, we subtract the intensity of one camera from the difference in the average intensity so that the intensity offset of the two cameras is equalized.
- Contrast Offset Adjustment
  After intensity offset equalization, the histogram centroid will be at the same intensity level, but the maximum and minimum intensity in the images captured by the two cameras may be the different. This results in a contrast difference. To ensure that the cameras have the same contrast level, we perform a contrast stretching procedure [32] such that the maximum and minimum of the intensity are the same in both cameras. As a result, the contrast offset is equalized.

#### 2.1.3. Mosaicking Process

**Figure 2:**
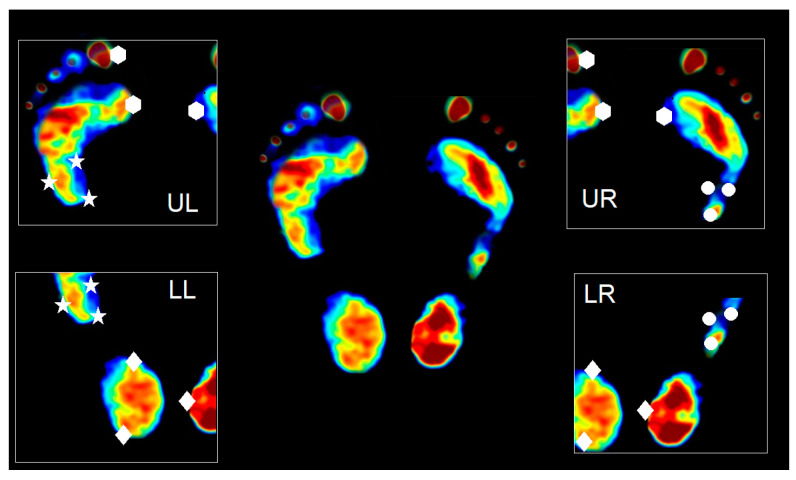
The four images captured from four cameras, which are denoted as UL, UR, LL, and LR.

The mosaicking process is used to stitch the multiple images acquired from multiple cameras into one full image. In our system, one foot will be captured by two cameras. As a result, four cameras are used for capturing the two-foot image. The four captured images will be denoted UL, UR, LL, and LR, as shown in Figure 2. To stitch the image, we use the landmark-based technique of image registration [33]. We first stitch UL with LL and UR with LR by manually selecting the landmark, which is also shown in Figure 2 as star and circle landmarks, respectively. With the corresponding landmarks, the geometric transform can be estimated and then used for stitching UL with LL and UR with LR. The process is repeated to stitch the two registered images to derive the full image of two feet shown in the middle of Figure 2.

#### 2.1.4. Smoothening Transition Region

**Figure 3:**
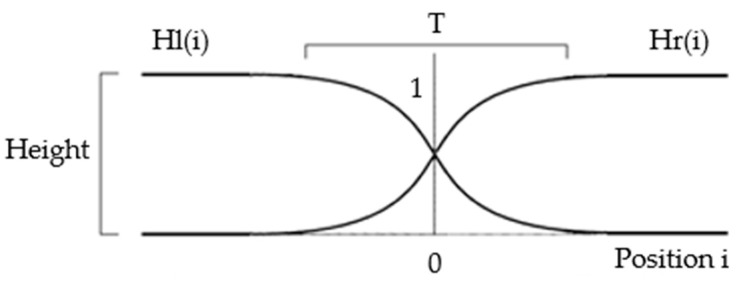
Weighted function for the smoothing of the transition region.

Despite equalizing the intensity and contrast of the captured image, a noticeable junction, appearing as a line, in the stitched image still exists. To resolve this problem, we use two hyperbolic weighted functions, as shown in Figure 3. We denote them as Hr(i) and Hl(i). Before the stitching of the two images, we multiply the horizontal (or vertical) intensity profile of the first image with the Hl(i) and the corresponding horizontal (or vertical) intensity profile of the second image with Hr(i). The addition of the weighted intensity profiles would yield the smoothed transient region in the stitched image.

### 2.2. Calibration 

**Figure 4:**
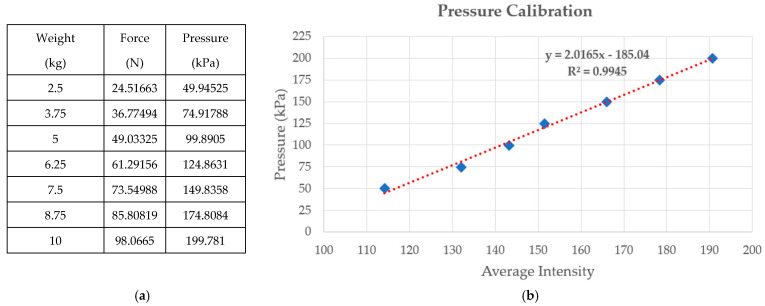
(**a**) A set of known weights and the associated computed forces and pressures; (**b**) plot of average intensity and pressure.

To validate the linear response of our pressure sensor, we design an experiment by placing a series of known weights on the optical-based plantar pressure measurement system, as shown in the first column of Figure 4a. The weight *m* is a cylindrical metal rod and hence generates a circular shape in the color-coded pressure image. The force and pressure can be computed by *F = mg* and *P = F/A*, where *g* is the specific gravity constant and *A* is the base area of the cylindrical metal rod. The plot of average intensity in the circular shape and the pressure is shown in Figure 4b, which demonstrates a linear response with R^2^ equal to 0.9945.

### 2.3. Foot Plantar Pressure Acquisition 

**Figure 5:**
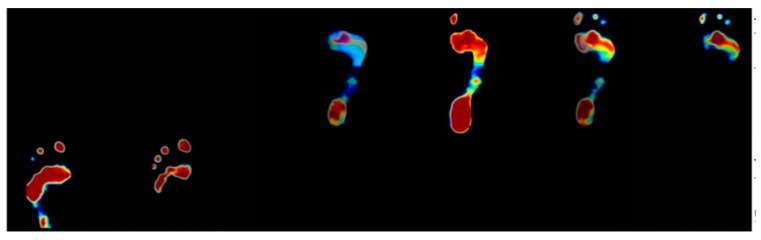
Series of captured images of gaiting foot plantar pressure acquisition. The left side is the plantar pressure when the subject starts to step onto the platform. The middle is the plantar pressure when the subject completely steps on. The right side is the plantar pressure when the subject steps away from the platform.

Our proposed optical-based foot plantar pressure acquisition can be applied for both static and gaiting plantar pressure modes. In the static mode, still images are captured by a series of cameras and stitched to derive the plantar pressure of the subject while standing. In the gaiting mode, the video is recorded by the series of cameras during the acquisition. The frame is then extracted. The corresponding frame is then stitched to derive the foot plantar for any moment in time. The stitched frame can then be displayed continuously to derive the gaiting plantar pressure of the subject while walking. Figure 5 shows a sample of gaiting plantar pressure images of the subject during walking instances. 

## 3. Human Posture Balance Measurement 

This subsection explains the application of our optical-based plantar pressure measurement for human posture balance evaluation. In human posture balance measurement, the subject usually stands on a measuring platform for some period, remaining as still as possible. The system then evaluates the efficiency of the standstill of the subject. Our system will measure the variation in the COP of the subject. A subject with high human-postural balance control efficiency would yield the minimum variation in COP [4,8]. We use the optical-based plantar-pressure measurement system with Visual Studio 2019 and MATLAB to display the foot pressure and COP of the subject. The subjects (*N* = 90) stand while wearing socks and relax on the platform for 1 min.

**Figure 6:**
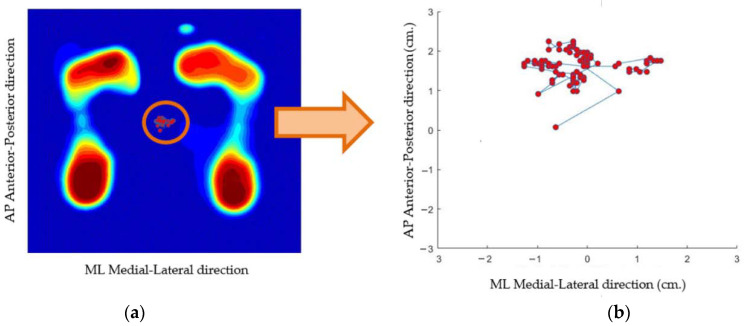
(**a**) COP trajectory is plotted superimposed with a foot pressure image. (**b**) COP trajectory is plotted in the 2D coordinate axis.

**Figure 7:**
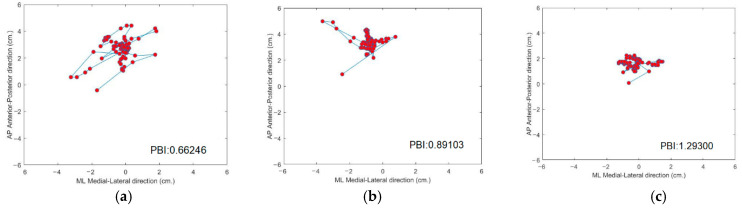
Trajectory of the COP of three subjects. (**a**) Trajectory of subject with weakest human posture balance; (**b**) trajectory of subject with medium human posture balance; (**c**) trajectory of subject with strongest human posture balance.

Our system represents the trajectory of the COP of the subject during a given time while wearing socks and relaxing. Figure 6 shows the result of the human postural balance analysis of the subject. In Figure 6a, a series of COP trajectories is plotted, superimposed with a foot pressure image. In Figure 6b, the COP trajectory is plotted in the 2D coordinate axis; the *x*-axis represents displacement in the medial–lateral direction, while the *y*-axis presents displacement in the anterior–posterior direction. The (0,0) coordinates are the image center. Samples of the COP trajectory of three subjects are shown in Figure 7.

Figure 7 shows the trajectory of the COP among three subjects. The COP trajectory of the first subject, (a) shown in Figure 7a, is scattered, which demonstrates that their human posture balance is weak. In contrast, the COP trajectory of the third subject (c), shown in Figure 7c, is congregated, which demonstrates that subject (c) has good human posture balance.

**Figure 8:**
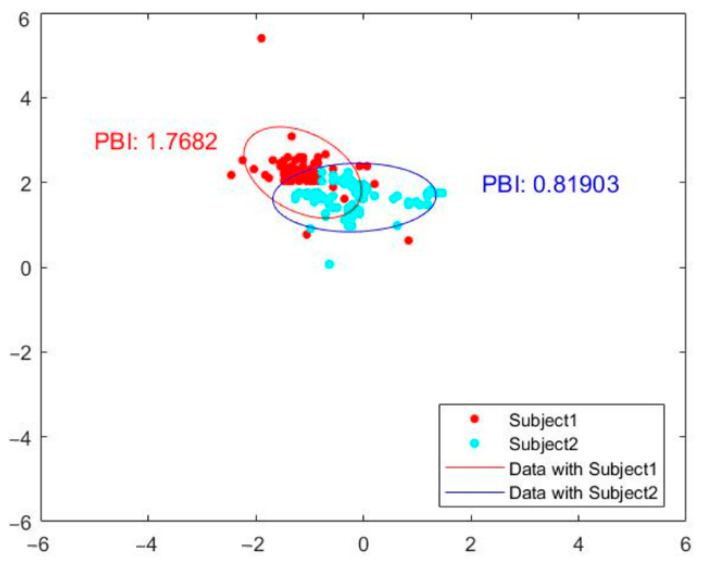
Ellipsoidal fit of COP trajectory labeled with posture balance index of two subjects.

To provide quantitative measurement for posture balance analysis using our optical-based plantar pressure measurement system, we compute the distance of the COP trajectory coordinate from the center. The coordinate points that are within the 95% percentile of the computed distance to the center will be used for ellipsoidal fitting. The inverse of the product of major and minor radius, defined as the posture balance index (PBI), can then be used as a static human postural balance evaluation. Subjects with better posture balance would have a higher PBI than a subject with poorer posture balance. Figure 8 shows the COP trajectory ellipsoidal fit labeled with the posture balance index of two subjects. The ellipsoidal fits of subjects 1 and 2 are shown as red and blue solid lines, respectively. The posture balance index (PBI) of subject 1 is 1.7682, whereas that of subject 2 is 0.81903. The PBI results indicate that subject 1 has better human postural balance than subject 2.

**Figure 9:**
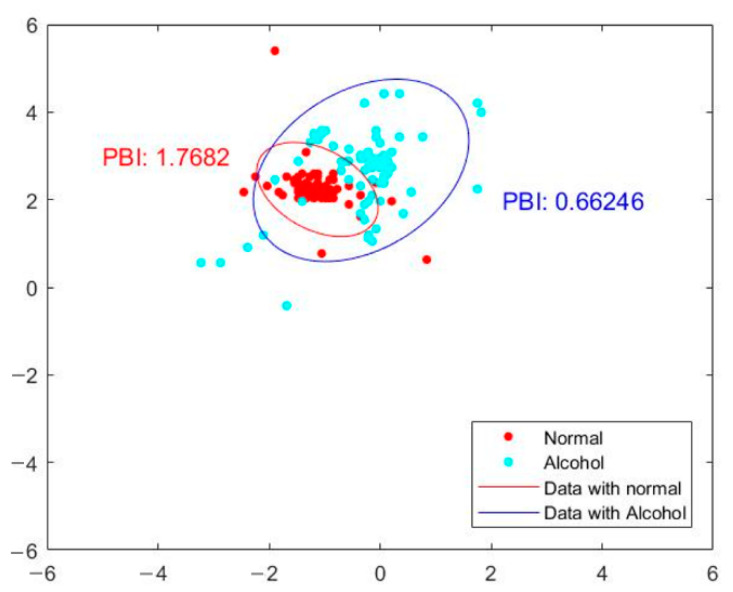
Posture balance index of a subject under the influence of alcohol (blue) compared with a normal subject (red).

We also demonstrate human posture balance measurement of subjects under various physical conditions—for example, a subject under normal conditions and a subject under the influence of alcohol. The result is shown in Figure 9. In the figure, the ellipsoid fit of the subject under the influence of alcohol is shown in blue. Their PBI is 0.66246. The ellipsoidal fit of the normal control subject is shown by the solid red line. The PBI of the normal subject is 1.7682, which is almost three times that of the subject under the influence of alcohol. In summary, the subject under the influence of alcohol displays a poorer posture balance index than the normal subject. Our proposed optical-based plantar pressure measurement system is hence capable of performing static foot plantar pressure evaluation (human postural balance) for subjects under various physical conditions.

## 4. Personal Identification and/or Recognition

Recently, the problem of personal verification and/or identification using footprint images has drawn considerable attention [22,23,24,25,26,27,34,35]. This subsection is devoted to the application of our optical-based plantar pressure measure system for personal identification and/or recognition. We will describe hybrid feature extraction, which includes static and gaiting plantar pressure features, the performance evaluation of identification and/or recognition, and the results of personal verification and/or identification.

### 4.1. Static Plantar Pressure Feature

We use the optical-based plantar pressure measurement system to classify human foot plantar according to three categories: normal foot, flat foot, and high arch. Flat foot is an abnormality of the foot plantar that usually is congenital. Individuals with flat foot always have an abnormal gait as they lack the recoiling mechanism of the foot during the gait cycle. The arch foot, on the other hand, involves a foot plantar that is abnormally curved. Individuals with arch foot also have an abnormal gait that is caused by the small contact area between the foot and the floor. Moreover, due to the bodyweight exerted on the small bearing area of the foot, inflammation and roughness of the skin occur. Without medical treatment, people with flat foot and high arch foot will eventually suffer from distortion of their skeleton, especially their spine. To classify the foot according to static plantar pressure feature, we defined an arch index value. The length of the foot in the vertical line is divided into equal thirds to give three regions: forefoot, mid-foot, and heel. Thus, before calculation, the foot pressure image must be rotated by a foot angle deviating from the vertical line. The arch index is calculated by dividing the mid-foot area by the total foot area, as shown in Equation (1).

(1) $$\mathrm{Arch}\;\mathrm{Index}=\frac{\mathrm{midfoot}}{\mathrm{forefoot}+\mathrm{midfoot}+\mathrm{heel}}$$

**Figure 10:**
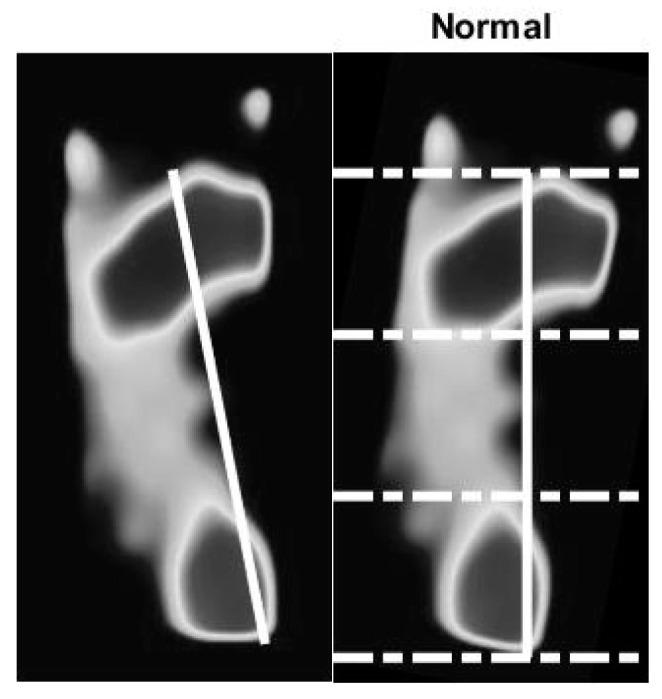
Normal foot with arch index of 0.23. Solid line is the principal axis of a normal foot. Dashed line divides the principal axis into equal distances. The ratio of the middle portion to the total foot area defines the arch index.

**Figure 11:**
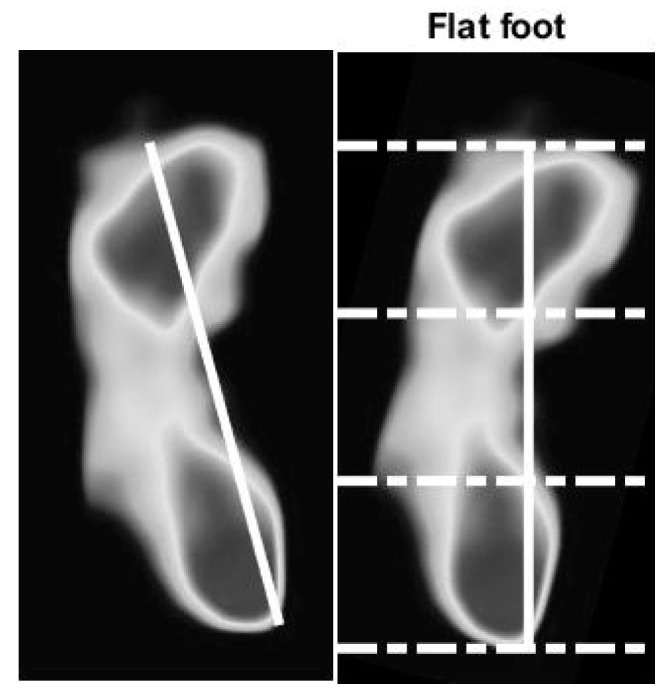
Flat foot with arch index of 0.31.

**Figure 12:**
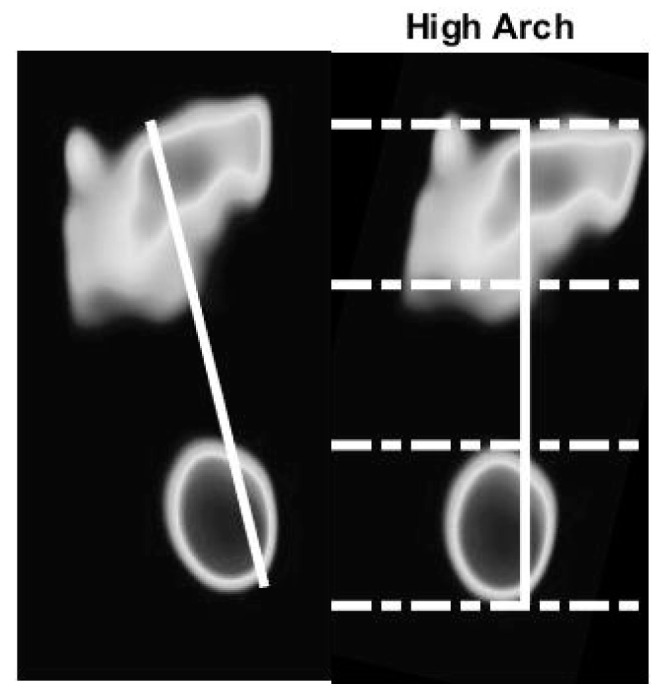
High arch foot with arch index of 0.16.

The flat foot has an arch index ≥0.26, the normal foot has an arch index between 0.21 and 0.26, and a high arch foot has an arch index ≤0.21 [36]. All subjects (*N* = 90) usually stand barefoot or while wearing socks on the platform for a moment. The footprint image is collected by this system. The essential parameter is then calculated and further used to classify the foot type. A sample of results of the static pressure measurement evaluation is shown in Figure 10, Figure 11 and Figure 12 for normal foot, flat foot, and high arch foot, respectively. It shows a sample of different foot types. In the figure, solid lines represent the principal axis, while dashed lines equally divide the principal axis and hence the foot plantar pressure into three equal spaces. Then, the arch index can be computed using Equation (1). The arch index for plantar pressure in Figure 10, Figure 11 and Figure 12 is 0.23, 0.31, and 0.16, respectively. The arch index will be used as one of the static features for person identification and/or recognition.

### 4.2. Gaiting Plantar Pressure Feature

In this section, we aim to use the system for gaiting plantar pressure feature extraction. The gaiting pressure analysis can be performing by averaging the plantar pressure over a period of time, recorded by the image while the subject walks on the platform for three steps using our optical-based plantar pressure measurement system. The platform is buried in the ground, such that the floor is level with the top of the platform. The subjects usually walk on the platform for a period of time. The video is recorded. The series of the extracted footprint image frames is then used to calculate the gaiting plantar pressure variation.

**Figure 13:**
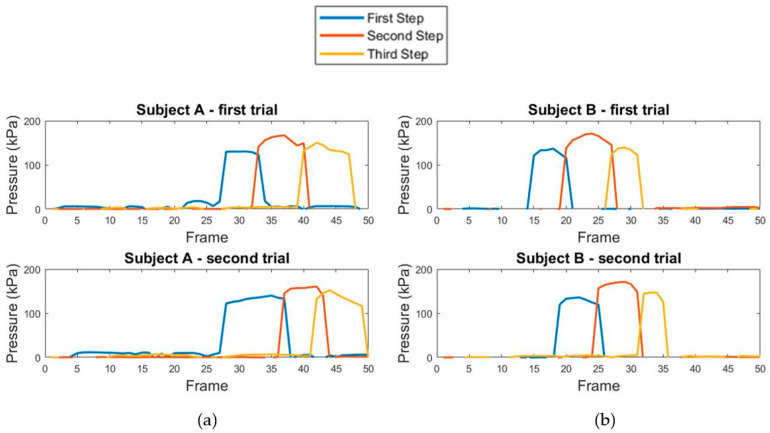
Graph of pressure variation over a period of time for two subjects (**a**,**b**) (left and right graph). Two acquisitions per subject (upper and lower graph).

The gaiting plantar pressure represents pressure variation used to analyze the subject’s walking pattern. We tested the gaiting plantar pressure acquisition with the subjects shown in Table 1. Figure 13a shows the pressure variation over a period of time from three steps in one subject. Figure 13b shows pressure variation over a period of time from another subject. The upper and lower graph present the pressure variation over a period of time in two gaits. The three traces in each graph are the pressure variation graphs for each step. Evidently, the upper and lower graph in Figure 13a,b show similarity in the pressure variation graph for the same subject, and this can be later used for gait biometrics. To measure the similarity of the pressure variation graph, the correlation coefficient [37] can be used. The coefficient value is between 1 and −1. A value close to one demonstrates high similarity between the two graphs. 

From Figure 13a, the correlation coefficient between the upper pressure variation graph (first trial) and the lower pressure variation graph (second trial) of subject A is 0.7204. From Figure 13b, the correlation coefficient between the upper pressure variation graph (first trial) and the lower pressure variation graph (second trial) of subject B is 0.8492. We then investigated the similarity between the pressure variation graphs across the subjects. The correlation coefficient between the upper pressure variation graph (first trial) of subject A and the lower pressure variation graph (second trial) of subject B is −0.1479. The correlation coefficient between the lower pressure variation graph (second trial) of subject A and the upper pressure variation graph (first trial) of subject B is −0.1671. We then confirm the uniqueness of the pressure variation graph, which can hence be used as one of the dynamic features for person identification and/or recognition.

### 4.3. Personal Identification and/or Recognition Evaluation 

Our proposed optical-based plantar pressure measurement system can be used for personal identification and/or recognition. We will demonstrate that several features can be extracted from the gait pattern and can be used for biometrics. The features can be categorized into static and dynamic features. The static features include the plantar pressure pattern (arch index) and the angle and the ratio of the major and minor axis of the ellipsoidal fit to the plantar pressure. The dynamic features include the step angle, step width, and graph plantar pressure variation over time. 

We use the optical-based plantar pressure measurement system for recording the gait in three steps. The data were collected from 90 individuals (Table 1). Each subject was measured over five trials of walking. We fused the pressure image time series for computing the static and dynamic features to use in personal identification.

**Figure 14:**
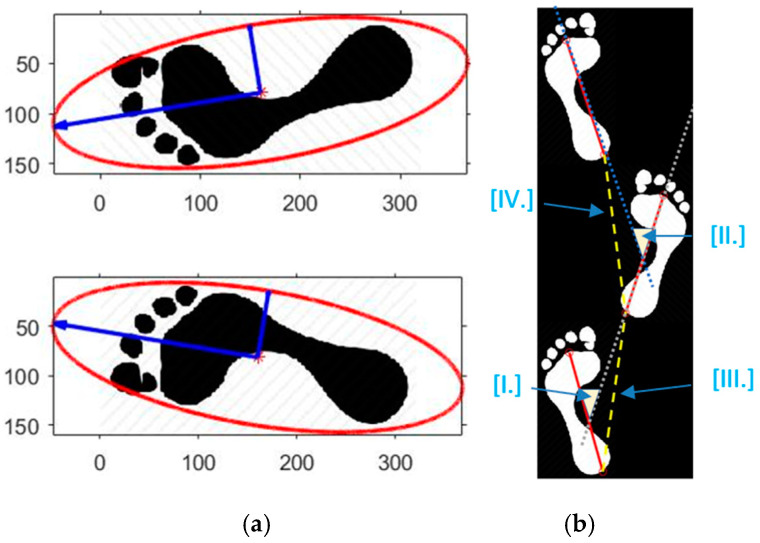
Illustration of the static (**a**) and dynamic feature (**b**). In (**a**), blue vectors are the two-principal axis, the red is the ellipsoidal fit; in (**b**), step angle (III–IV) is the angle between the principal axes of the left and the right foot. Step width (I–II) is defined to be the distance between each step of the subject.

The static features include the arch index, foot angle, and the ratio of the major and minor axis of the ellipsoidal fit. The arch index is the fundamental feature used to classify the foot into normal foot, flat foot, or arch foot. To determine the arch index, we calculate the principal foot axis using ellipsoidal fitting of the foot pressure image, as shown n Figure 14a. To improve the robustness to noise, we perform the ellipsoidal fit only for 95% of the plantar pressure data, which is within two standard deviations from the mean. The length of the principal axis line joining the highest point and the lowest point is then divided into three equal segments. The foot pressure image is then divided into three parts spanning each segment. The area of the middle portion to the total portion is then defined as the arch index. A subject with a flat foot is likely to have a large middle portion and hence a high arch index, and vice versa. Foot angle is defined as the principal angle of the foot with the vertical axis. In summary, the static features vector of subject *i*^th^ ($Fs_{i}$) is defined as follows:

(2) $$Fs_{i}=\left[\alpha_{i1},\propto_{i2},\propto_{i3},\right]$$

where $\propto_{i1}$ is the arch index of the *i*^th^ subject defined in Equation (1), $\propto_{i2}$ is the foot angle of the *i*^th^ subject, and $\propto_{i3}$ is the ratio of the major and minor axis of the ellipsoidal fit to the static foot pressure image of the *i*^th^ subject. 

The dynamic features including step angle and step width are shown in Figure 14b, denoted as I-II and III-IV, respectively. The angle between the principal axis of the left and the right foot is accounted for in the step angle feature. Step width is defined to be the distance between each step of the subject. It can be determined by the length between the corresponding point in each foot pressure image at the lowest point. The dynamic feature vector of subject *i*^th^ ($Fd_{i}$) is written as follows:

(3) $$Fd_{i}=\left[\beta_{i1},\beta_{i2},\beta_{i3}\right]$$

where $\beta_{i1}$ is the step angle of the *i*^th^ subject and $\beta_{i2}$ is the step width of the *i*^th^ subject. The other dynamic feature $\beta_{i3}$ that can be used for person recognition is the similarity (correlation coefficient) of the pressure variation graph as described in Section 4. 

The total feature vector $S_{i}$ is defined as the combination of dynamic feature vector ($Fd_{i}$) and static feature vector ($Fs_{i}$):

(4) $$S_{i}=\left[Fd_{i},Fs_{i}\right]$$

Features *S_i_* are then used to calculate the matching score and create the ROC curve. The area under the ROC curve can represent the efficiency of our system for personal recognition. To define the matching score, we computed the error function of the matching pair with Equation (5), where $s_{i}$ and $t_{i}$ are *i*^th^ feature vector of subject *s* and *t*, respectively, and *N* is the number of features used for calculation. The error function is defined as

(5) $$Error\left(\tilde{E}\right)=\frac{\sum_{i=1}^{N}{\left(s_{i}-t_{i}\right)}^{2}}{N}$$

The matching score $\tilde{M}$ or similarity score of testing can then be calculated from $\;error\left(\tilde{E}\right)$ as

(6) $$\mathrm{Matching}\;\mathrm{Score}\left(\tilde{M}\right)=1-\tilde{E}$$

**Figure 15:**
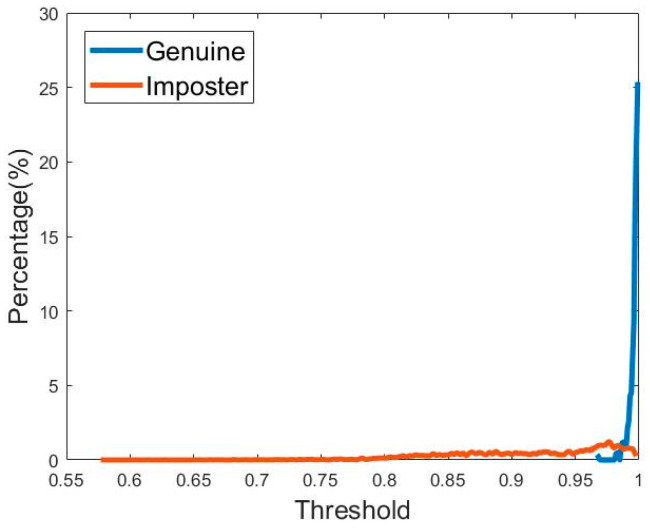
Distribution of genuine and imposter matching score. Genuine distribution is shown in blue whereas imposter distribution is shown in red. The intersection of genuine distribution and imposter distribution is the equal error rate (ERR).

**Figure 16:**
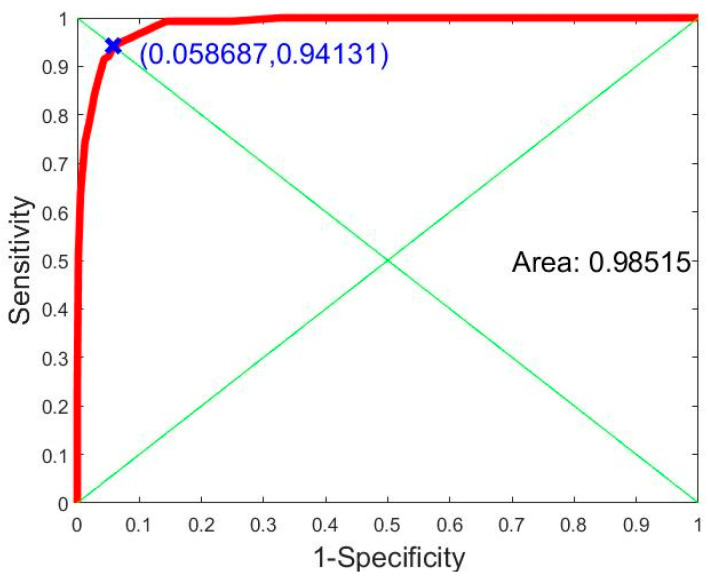
ROC curves showing area under the ROC curve of 0.98396.

As a result, the histogram distribution of the matching score function for genuine matching and imposter matching are shown in Figure 15. A receiver operating characteristic (ROC) curve is presented in Figure 16. This curve was created from the histogram distribution with specificity and sensitivity within any threshold. 

The *x*-axis of the ROC curve shows specificity (false match rate) and the *y*-axis shows sensitivity (true match rate). These points on the ROC curve represent the specificity and sensitivity of the threshold in the histogram distribution. The area under the ROC curve is 0.98515 (98.515%). This can be used to measure the recognition rate or efficiency of the system for person identification. Another indicator used in person identification is the equal error rate (EER) [38], the point on the ROC curve at which the true match rate and false match rate are equal. This point is obtained by intersecting the ROC curve with a diagonal of the unit square. According to the ROC curve in Figure 16, our plantar pressure biometric system can achieve an EER of 5.8687%.

## 5. Discussion

This research concerns the design and construction of an optical-based foot plantar pressure measurement system for application in person identification and/or recognition. There are a number of issues that need to be addressed for the completeness of our research proposal, which are as follows.

(i) In the proposed technique of person identification and/or recognition, we have defined the total feature vector *Si* as the combination of the dynamic feature vector ((*Fd*}*_i_*) and static feature vector ({*Fs*}*_i_*). In practice, the combination can be weighted as
  *Si* = *α* (*Fd*}*_i_* + (1 − *α*) {*Fs*}*_i_* (7)
  where *α* is between 0 and 1. The value of *α* determines the weight of the feature vector. If *α* is equal to 0.5, the contributions of the dynamic feature vector and static feature vector are equal. If *α* > 0.5, the contribution of the dynamic feature vector is greater than that of the static feature vector, or vice versa. The *α* > 0.5 is used when we need to improve the specificity of the system as the dynamic feature is more robust than the static feature. In our experiment, we set *α* = 0.5.
(ii) Our optical-based plantar pressure measurement system uses multiple USB cameras to capture foot plantar images. The height of the measurement platform depends on the focal length of the USB camera. Using the OKER 386 USB camera, the height of the platform is approximately 18 cm. The top surface of the system, which is not level with the nearby floor, makes the system inconvenient to use. To alleviate this problem, a wooden platform was built surrounding our measurement system such that the top surface of our system is level with the top of the wooden platform.
(iii) One unit of our optical-based plantar pressure measurement system requires eight USB cameras and can measure gaiting plantar pressure for up to three steps. The active area of the measurement system is 30 cm × 72 cm. The number of pixels in the active area is 600 pixels × 1440 pixels. The resolution of our system is hence 400 pixel/cm^2^. The resolution of our optical-based plantar pressure measurement system is much higher than that of a commercial pressure sensor mat, which has an average resolution of only 25 sensor/cm^2^ [39]. To speed up the acquisition process, our proposed system can decrease the resolution to 200 pixel/cm^2^. Nevertheless, the resolution is still higher than that of the commercial system. In the event that a greater active area is needed, our measurement system can be concatenated to create a longer walkway. This makes our system expandable and its construction affordable.
(iv) To achieve the best performance, our system is designed to be used with a barefoot subject. In the case that the subject is wearing shoes, the robustness of the static feature will be degraded. In such a case, the *α* in Equation (7) can be adjusted to 1 and only the dynamic feature is included in the total feature.
(v) All the digital image processing used in our optical-based plantar pressure measurement system is based on OpenCV library [40]. The OpenCV digital image processing can run on many platforms, e.g., a personal computer with a Windows operating system or a Raspberry Pi microcontroller with the LINUX operating system. A personal computer provides the best performance, with a fast acquisition process. The Raspberry Pi microcontroller can be used for a portable system. Our system uses the number of USB cameras connected to personal computer or Raspberry Pi microcontroller. A USB hub is required. In general, the frame rate of a typical USB camera is 30 frames per second (fps) which is sufficient for gaiting plantar pressure acquisition. In the case of using a USB hub, however, the frame rate will be decreased to some extent as the bandwidth of the USB port is shared among all connected cameras. The problem, however, can be lessened using USB 2.0.
(vi) The application of our optical-based plantar pressure measurement for static plantar pressure measurement including human postural balance and foot classification can be performed in real time. For the person recognition/identification application, since it requires the acquisition of the gaiting plantar pressure feature, which requires the subject to walk for three steps, there will be a delay of a few seconds in reporting the recognition/identification results. In addition, the static plantar pressure image is captured when subject fully steps onto the platform. The area of the plantar pressure image is the maximum.
(vii) The overall person identification performance using plantar pressure features is promising, with a recognition rate of 98.396% and an equal error rate (EER) of 6.5808%. Compared with Connor [27], who also has studied the underfoot pressure features for barefoot gait biometrics on a dataset of 92 subjects, our proposed method achieves a better result on a walking dataset of 90 subjects. In Connor’s work, a recognition rate of 93% is reported. Our proposed algorithm’s person identification performance achieved a recognition rate similar to that of Pataky’s work [41], which also evaluated the performance of a large number of barefoot subjects. Both Connor’s and Pataky’s data were recorded on a commercial pressure mat with a fine resolution of approximately 5 mm. Our optical-based pressure sensor provides a finer resolution of 0.5 mm, which is ten times greater than the commercial mat used by Connor and Pataky. Using a commercial pressure mat that provides a sampling rate of 100 Hz, Connor’s and Pataky’s data include both a normal walking pace and a fast pace. Our proposed method relies on multiple USB cameras, where the USB bandwidth has to be shared, so it can study only the normal walking pace dataset. Compared with a non-plantar-pressure-based system, our personal identification system can also achieve a higher personal identification accuracy than that of Sinha et al. [22], which uses Kinect to perform area and distance feature recognition, with a recognition rate of 90%. Personal identification from the three-dimensional range information of the target, as performed by Yamada et al. [23], used a real-time multi-line light detection and ranging (LiDAR) camera and the collected data were trained and classified by a convolution neural network, with a recognition rate of only 60%. In addition, Uhl and Wild [42] designed a footprint-based feature extraction system including geometry, shape, and texture, with a recognition rate of 85%.
(viii) Our human posture balance measurement system is based on plantar pressure measurement, which is similar to the work of Hernandez et al. [4] and Gopalai et al. [8]. Hernandez et al. [4] used a ground-level six-channel force plate (AccuGait, AMTI, Watertown, MA, USA) to measure the human posture balance of 11 subjects. The data were collected in various situations, including with eyes open, eyes closed, bipedal, and unipedal stance. To estimate human posture balance, Hernandez et al. used the COP, velocity autocorrelation function (COP-VAF), and stabilogram diffusion analysis (SDA). Due to the intensive data processing, the posture balance results were reported off-line after collecting and processing data for a period of time. Compared with the work of Hernandez et al., the resolution of our human posture balance system is higher, while our sampling rate is poorer. Human posture balance measurement, however, does not require a high sampling rate, which makes our optical-based human balance system feasible to be used for human posture balance measurement. Our human posture balance index (PBI) is based on an ellipsoidal fit to the trajectory of COP. The advantage of PBI is that the index contains both magnitude and direction. The magnitude of the major and minor axis of the fitted ellipsoid reflects the magnitude of human posture balance, while the principal axis of the ellipsoid reflects the direction. In term of data collection, Hernandez et al. collected data for more possible factors, which is required for human posture balance evaluation. Although we collected data for a large number of subjects and also included a subject under the influence of alcohol, we need further studies to test the reliability of the system for other factors affecting human balance control. In Gopalai et al.’s [8] work, eighteen force-sensing resistors (FSR) were used to measure human posture balance in 18 subjects. The COP was used for human posture balance evaluation. Both our system and that of Gopalai et al. are capable of real-time measurement. The 40 mm resolution of Gopalai et al.’s measurement system is, however, significantly lower than that of our optical-based measurement system.

## 6. Conclusions

In this research, we propose an optical-based plantar pressure measurement system. The system consists of a series of digital cameras installed underneath an acrylic-top platform. Due to the pressure exerted between the glossy white paper installed at the top of the acrylic and the acrylic plate, the light of the LED strip mounted along the side of the acrylic plate is deflected towards the digital cameras and provides a color-coded plantar pressure image of the subject standing on the platform. The captured color-coded plantar pressure images from each camera are mosaicked to increase the measurement area, which can be applied for dynamic measurement. We use a series of USB cameras aligned under the acrylic-plate walking platform. The captured images are then concatenated with the mosaic image processing in order to increase the sensing area. Capturing image data in video mode, various dynamic parameters can be derived for further dynamic analysis. The images captured in one specific frame can be used for static analysis. 

Our proposed plantar pressure measurement system has three major advantages. Firstly, our proposed plantar pressure measurement system is a real-time, low-cost, and easy-to-implement system that can be applied for human postural balance and person recognition/identification system. Secondly, the system is a marker-free and sensor-free setup for human postural balance and person recognition/identification. As a result, the system does not obscure the national motion of the human body. Thirdly, the system can provide both static features and dynamic features for person identification and/or recognition. 

To perform static evaluation using our optical-based plantar pressure measurement system, the subject stands on the platform while the COP is computed and displayed in real time. For dynamic evaluation, a graph of pressure of both the left and right foot is plotted to evaluate the gait balance of the subject. This results in biometric data for both static and dynamic plantar pressure, which can be used for personal identification and/or recognition, demonstrating promising results. Furthermore, our foot plantar identification system can achieve an area under the ROC curve of 0.98515 (98.515%) and an equal error rate (EER) of 5.8687%. However, there are two major limitations in our optical-based plantar pressure measure system. The first limitation is that it can measure only three walking steps; thus, identification of persons with a longer step width remains to be taken into account. The second limitation is that our optical-based plantar pressure measurement system requires stable light intensity from LED. Therefore, a power stabilizer is needed for the system. 

## Figures and Tables

**Table 1:** Subject data. Average with standard deviation.

	Female	Male
n	50	40
Age (years)	20.98 (1.778)	21.575 2.934)
Mass (kg)	54.7 (8.758)	69.2 (8.618)
Height (cm)	160.02 (5.192)	173.075 (6.455)

## Data Availability

Not Applicable.
